# RNA-Seq Reveals Function of Bta-miR-149-5p in the Regulation of Bovine Adipocyte Differentiation

**DOI:** 10.3390/ani11051207

**Published:** 2021-04-22

**Authors:** Hongfang Guo, Rajwali Khan, Sayed Haidar Abbas Raza, Syed Muhammad Suhail, Hamayun Khan, Sher Bahadar Khan, Ayman Hassan Abd El-Aziz, Linsen Zan

**Affiliations:** 1 Medical College, Xuchang University, Xuchang, Henan 461000, China; guohongfangkl@126.com; 2 Livestock Management, Breeding and Genetics, The University of Agriculture, Peshawar 25120, Khyber Pakhtunkhwa, Pakistan; smsuhail@aup.edu.pk (S.M.S.); hamayunkhan@aup.edu.pk (H.K.); sbvetdr@yahoo.com (S.B.K.); 3 College of Animal Science and Technology, Northwest A&F University, Yangling 712100, China; haiderraza110@nwafu.edu.cn; 4 College of Veterinary Sciences, The University of Agriculture, Peshawar 25120, Khyber Pakhtunkhwa, Pakistan; 5 Department of Animal Husbandry and Animal Wealth Development, Faculty of Veterinary Medicine, Damanhour University, Damanhour 22511, Egypt; ayman.sadaka@vetmed.dmu.edu.eg; 6 National Beef Cattle Improvement Research Center, Yangling 712100, China

**Keywords:** bta-miR-149-5p, bovine adipocytes, lipid metabolisms, RNA-Seq

## Abstract

**Simple Summary:**

This is a novel study that explored the function of bta-miR-149-5p in the lipid metabolism of bovine adipocytes. Previously, we explored the role of bta-miR-149-5p gene in proliferation and differentiation of bovine adipocytes; however, the underlying mechanism of bta-miR-149-5p function in the regulation of lipid metabolism and adipogenesis in bovine adipocytes is unexplored. A transcriptomic study with integration of bioinformatics study was performed to fully explore the function of bta-miR-149-5p microRNA in bovine adipogenesis. This study will help the scientific community deal with lipogenesis for the breed improvement program of cattle for the provision of healthy meat to consumers.

**Abstract:**

Intramuscular fat is a real challenge for the experts of animal science to improve meat quality traits. Research on the mechanism of adipogenesis provides invaluable information for the improvement of meat quality traits. This study investigated the effect of bta-miR-149-5p and its underlying mechanism on lipid metabolism in bovine adipocytes. Bovine adipocytes were differentiated and transfected with bta-miR-149-5p mimics or its negative control (NC). A total of 115 DEGs including 72 upregulated and 43 downregulated genes were identified in bovine adipocytes. The unigenes and GO term biological processes were the most annotated unigene contributor parts at 80.08%, followed by cellular component at 13.4% and molecular function at 6.7%. The KEGG pathways regulated by the DEGs were PI3K-Akt signaling pathway, calcium signaling pathway, pathways in cancer, MAPK signaling pathway, lipid metabolism/metabolic pathway, PPAR signaling pathway, AMPK signaling pathway, TGF-beta signaling pathway, cAMP signaling pathway, cholesterol metabolism, Wnt signaling pathway, and FoxO signaling pathway. In addition to this, the most important reactome enrichment pathways were R−BTA−373813 receptor CXCR2 binding ligands CXCL1 to 7, R−BTA−373791 receptor CXCR1 binding CXCL6 and CXCL8 ligands, R−BTA−210991 basigin interactions, R−BTA−380108 chemokine receptors binding chemokines, R−BTA−445704 calcium binding caldesmon, and R−BTA−5669034 TNFs binding their physiological receptors. Furthermore, the expression trend of the DEGs in these pathways were also exploited. Moreover, the bta-miR-149-5p significantly (*p* < 0.01) downregulated the mRNA levels of adipogenic marker genes such as CCND2, KLF6, ACSL1, Cdk2, SCD, SIK2, and ZEB1 in bovine adipocytes. In conclusion, our results suggest that bta-miR-149-5p regulates lipid metabolism in bovine adipocytes. The results of this study provide a basis for studying the function and molecular mechanism of the bta-miR-149-5p in regulating bovine adipogenesis.

## 1. Introduction

Adipose tissue stores energy in the form of neutral triglycerides and performs a vital function in maintaining energy homeostasis [1]. Adipocytes are the primary cell types of adipose tissue. There are two ways of adipose tissue development: one is hypertrophy (increase in adipocyte cell mass) and other is hyperplasia (increase in adipocyte numbers). In the case of surplus energy, the adipocytes deposits triglycerides (lipid droplets) through the lipogenic pathway, and in the case of energy deficiency, it releases lipids through the lipolysis pathway [2,3]. Mammalian body contains four important fat depots, namely, visceral, subcutaneous, intermuscular, and intramuscular (IM) fat. In livestock species, the IM fat is considered one of the most important factors that determines carcass quality traits. However, selective improvement of IM fat without altering the other three adipose depots is very challenging in the meat industry [4]. The IM fat deposition contributes to the development of meat sensory qualities such as marbling, juiciness, flavor, and tenderness [5]. Hence, approaches for the improvement of IM fat deposition are crucial for the development of meat quality. Therefore, research needs to be conducted to explore the underlying mechanisms of adipogenesis for the improvement of meat quality [6]. 

miRNAs are a class of endogenous small non-coding, approximately 22 nt, single-stranded RNAs, and play a role in post-transcriptional regulation by targeting mRNA degradation or inhibiting their translation [7]. A single miRNA can target hundreds of potential genes that can integrate complex molecular regulatory networks [8,9]. miR-143 was the first identified, with a proven role in adipocyte differentiation by regulating their target gene ERK5 (extracellular signal-regulated kinase 5) [10]. Previously, a large number of miRNAs were identified with their proven roles in regulation of adipose proliferation and differentiation. For instance, miR-2400 promoted bovine preadipocyte proliferation by directly targeting PR/SET domain 11 (PRDM11) [11]. The regulatory roles of miR-145 were determined in bovine adipogenesis through PI3K/Akt and MAPK signaling pathways [12]. Moreover, miR-144-3p suppresses preadipocyte proliferation [13]. The role of miR-424 in bovine adipogenesis through direct regulation of serine/threonine kinase 11 (STK11 also known as LKB1) was also identified through regulation of AMP-activated protein kinase (AMPK) [14]. Similarly, miR-17-92, miR-27, miR-93, miR-130, and miR-378 are key regulators of adipogenesis through adipocyte proliferation or differentiation [15,16,17,18]. Previously, our group exploited the negative regulatory role of miR-23a–miR27a–24-2 cluster in bovine adipocyte differentiation [19], and bta-miR-130a-b was found to affect bovine adipocyte differentiation by targeting PPARγ and CYP2UI [20]. Recently, studies have shown that miR-149 plays a pivotal role in the pathogenesis of various types of carcinomas including cancers of the digestive system [21], colorectal cancer [22], hepatocellular cancer [23,24], cancer of the respiratory system [25], breast cancer [26], and sebaceous gland carcinoma of the eyelid [27]. Therefore, this microRNA may be used as a potential diagnostic marker and therapeutic target against cancers. The miR-149-3p regulates energy homeostasis through the PRDM16 gene in visceral adipocytes [28]. Remarkably, miR-149 suppresses osteosarcoma through inhibiting TWEAK-Fn14 axis and is used as a potential therapeutic agent in osteosarcoma patients [29]. Interestingly, miR-149 enhances mitochondrial biogenesis and its activity through PGC1α activation by suppressing PARP-2 poly (ADP-ribose) polymerase-2 gene and enhancing cellular NAD+ levels through activation of the SIRT-1 gene. In obesity, miR-149 regulates mitochondrial biogenesis and function in SIRT-1/PGC-1α pathway through PARP-2 gene, and also regulates proliferation and differentiation of follicular B cells [30]. These findings proved the regulatory role of miR-149 in type-2 diabetes, high-fed diet-induced obesity, cancers, and cardiovascular diseases [31]. Previously, we proved the role of bta-miR-149-5p in bovine adipocyte proliferation and differentiation through regulation of TORCs [32]. It is intriguing that the regulatory function of bta-miR-149-5p will expand our understanding about the molecular mechanisms of bovine adipogenesis, which can be applied to enhance meat quality grading.

## 2. Materials and Methods

### 2.1. Ethical Statement

The experimental animals were dealt with as per standard operating procedures (SOPs) formulated by the Chinese Council of Animals Care, and further approved by “Experimental Animal Management Committee (EAMC)” of the Northwest Agricultural and Forestry University, notified vide notification no. EAMC/20-23 dated 20.04.2013. The sampling was performed after human killing of the experimental animals at the National Beef Cattle Improvement Research Center, Yangling, Shaanxi, China.

### 2.2. Isolation of Bovine Primary Preadipocytes

Bovine preadipocytes were isolated from healthy newborn calves (5 days old) of Qinchuan cattle by using previously described methods [19,33,34,35,36,37]. The longissimus dorsi muscle area was exposed and extracted under aseptic conditions, and the muscle was first cleaned with 75% ethanol and then incised with the help of a sharp sterile surgical curved scissors. The adipose tissue samples were washed with PBS containing 10% penicillin/streptomycin and immediately transferred to the cell culture room for further processing. The tissues were separated from the blood vessels and connective tissues under a stereo dissecting microscope with the help of sterile forceps. The adipose tissues were then crushed and subjected to enzyme digestion with collagenase I, 0. 25% (Sigma, Shanghai, China) for 1 h at 37 °C in a shaking water bath. The digestive mixture sample was then neutralized with equal volume of 10% FBS (Invitrogen, Carlsbad, CA, USA) and strained by using 100 μm and then 40 μm strainers. The filtrate was centrifuged at 1500 × *g* for 10 min, and the cell pellet was washed 2 times with DMEM-F/12 medium (Gibco, Grand Island, NY, USA) without serum. The isolated cells pellet was suspended within DMEM-F/12 medium supplemented with 10% FBS, then seeded in 60 mm collagen-coated cell culture plates, and finally incubated at 37 °C and 5% CO_2_ for 1 h. The medium was changed and then washed 3 times with PBS to remove the debris and free-floating dead cells.

### 2.3. Transient Transfection, Cell Differentiation, and Staining for Lipid Droplets

The bovine adipocyte cells were seeded in 24-well plates and cultured in DMEM-F/12 (Dulbecco’s modified Eagle’s medium; Gibco, Grand Island, NY, USA) with 10% FBS (Invitrogen, USA) and 1% antibiotics (100 IU/mL penicillin and 100 μg/mL streptomycin), and incubated at 37 °C with 5% CO_2_. The cells were transiently transfected with bta-miR-149-5p mimic (50 nM) and mimic negative control (50 nM) at 70–90% confluence and density of 1.2 × 105 cells with growth medium without antibiotics and FBS using lipofectamine 3000 transfection reagent (Invitrogen, CA, USA). Briefly, the RNA oligonucleotides and the transfection reagent were diluted separately in Opti-MEM medium (Gibco) and incubated for 10 min at RT. Both the reagents were mixed, combined, and incubated for another 15 min at RT to form the transfection reagent–RNA complexes. The complexes were then added dropwise to the cells and incubated for 24 h before changing to fresh medium. 

At 48 h post-transfection, the cells were differentiated with first differentiation media including 0.5 mM hydro cortisol, 1μM dexamethasone, IBMX (3. isobut-1-methylxanthine), and 167 nM insulin, as previously described [38]. After 2 days, the media was replaced with second differentiation medium (DMEM-F/12) supplanted with 10% fetal bovine serum and 167 nM insulin. The cells were fixed and stained with red oil O staining at the ninth day of differentiation. Briefly, the stock solution was prepared by dissolving red oil O staining powder in isopropanol under dark. The solution was filtered and diluted with 60% deionized water and re-filtered to prepare working solution. Before staining, the adipocytes were washed 3 times with 1X PBS and then fixed with 4% paraformaldehyde solution for 30 min at room temperature, then washed again with 1XPBS for 3 times. Then, the cells were stained with working solution of red oil O staining for 30 min at room temperature. The images were captured using an Olympus IX71 microscope (OLYMPUS), and lipid droplets were observed. The lipid droplets were also analyzed using BODIPY staining. The adipocytes were fixed and then incubated for 30 min at room temperature in a 1:1000 dilution of 1 mg/mL BODIPY 493/503 (Invitrogen) diluted with 1XPBS at room temperature. 4,6-Diamidino-2-phenylindole (DAPI) was used for the identification of the nuclei. Images were captures with a Live Cell Imaging System (Nikon Instruments, Europe BV, Kingston, Surrey, UK). ImageJ software (National Institutes of Health, Bethesda, MA, USA) was used for the quantification of lipid accumulation in adipocytes from the fluorescence intensity of the BODIPY stain (green fluorescence).

### 2.4. RNA Isolation, Construction of cDNA Library, and qRT-PCR 

After 48 h of adipocyte treatment with bta-miR-149-5p mimics or mimics NC (negative control), the adipocytes were collected and RNA was extracted through RNAiso Plus kit (Takara Beijing, China). The RNA integrity both quantity and quality were evaluated through optical density (OD) of 260 and ratio of the OD of 260/280 with Nano Quant plate TM (TECAN, Infinite M200 PRO). The RNA quality was further analyzed through 1% agarose gel. Then, the RNA was subjected to reverse transcription for cDNA synthesis through PrimeScriptTM RT reagent kit (perfect real time) with a gDNA eraser (Takara, Beijing, China). The qRT-PCR was performed using SYBR^®^ Premix Ex Taq II kit (Takara, Beijing, China). For the bta-miR-149-5p expression analysis, we used a mircute miRNA isolation kit (TIANGEN, Beijing, China). The first strand cDNA library was constructed using miRcute Plus miRNA First-Strand cDNA kit (TIANGEN, Beijing, China). The bta-miR-149-5p expression was measured through mircute plus miRNA qPCR detection kit (TIANGEN, Beijing, China). For gene expression analysis, GAPDH and β-actin were used, while for miRNA expression, U6 was used as housekeeping genes. The 2^−ΔΔCt^ method was used for the identification of relative mRNA and miRNA expression levels [39]. The PCR primer sequences are shown in Table S1.

### 2.5. RNA-Seq Library Construction, Quality Control, and Sequencing

The total RNA was extracted with Trizol reagent kit (Invitrogen, Carlsbad, CA, USA), and the RNA quality was analyzed through an Agilent 2100 Bioanalyzer (Agilent Technologies, Palo Alto, CA, USA). The quality was further assessed through RNase free agarose gel electrophoresis. After extraction of total RNA, Oligo (dT) beads were used for the enrichment of mRNA, and fragmentation buffer was used for making short fragments, and then reverse-transcribed into cDNA using random primers. The DNA polymerase I, dNTP, RNase H, and buffer were used for the synthesis of second-strand cDNA, and their fragments were purified using QiaQuick PCR extraction kit (Qiagen, Venlo, the Netherlands); poly(A) was added for end repair and then ligated into Illumina sequencing adapters. The ligation products were size-selected by agarose gel electrophoresis, PCR amplified, and sequenced using Illumina HiSeq2500 by Gene Denovo Biotechnology Co. (Guangzhou, China).

### 2.6. Bioinformatics Analysis

#### 2.6.1. Filtering of Clean Reads

After sequencing, the raw reads including adapters and low quality bases (reads containing more than 10% of unknown nucleotides “N” and the reads containing more than 50% of low quality bases “Q-value ≤ 20”) were filtered through fastp software version 0.18 [40]. 

#### 2.6.2. Alignment with Ribosome RNA (rRNA) and Reference Genome

The reads were mapped to ribosomal RNA (rRNA) database using the short reads alignment tool Bowtie-2 software version 2.2.8 [41]. Then, the rRNA-mapped reads were removed, and clean reads were used in gene abundance calculation. The reference genome index was created, and clear reads were mapped with reference genome through HISAT2. 2.4 software using “rna_strandness RF” and with all other parameters set as a default [42].

#### 2.6.3. Quantification of Gene Abundance

The fragment per kilobase of transcript per million mapped reads (FPKM) value was calculated for quantification and variations using StringTie software [43,44]. The FPKM formula is shown as follows:

$$FPkM=\frac{10^{6}c}{NL/10^{3}}$$

where *FPkM* (A) refers to the expression of gene A, “*c*” denotes the number of fragments mapped to gene A, “*N*” is the total number of fragments mapped with reference genes, and “*L*” is number of bases in gene A. 

#### 2.6.4. Deferentially Expressed Genes (DEG) Identification

The DEGs between the 2 groups was analyzed with DESeq2 software [45], and edgeR software was used for variation between the 2 samples [46]. The transcripts with FDR (false discovery rate) below 0.05 and absolute fold change with ≥2 were considered to be DEGs.

#### 2.6.5. GO Enrichment Analysis

The DEGs were mapped into GO terms through GO enrichment analysis using the GO database available at http://www.geneontology.org/ (accessed on 20 April 2021). The significantly enriched GO terms in the DEGs correspond to the respective biological functions. Hypergeometric test was used for the calculation of gene numbers for each term in comparison with the reference genome using the following formula:

$$P=1-\overset{m_{-1}}{\underset{i=0}{\sum }}\frac{\left(\begin{array}{c} m\\ i \end{array}\right)\left(\begin{array}{cc} N- & M\\ n- & i \end{array}\right)}{\left[\begin{array}{c} N\\ n \end{array}\right]}$$

where “*N*” denotes the number of all transcripts within GO annotation, “*n*” is the number of DEGs in *N*, “*M*” is the number of all transcripts annotated without respective GO terms, and “m” is the number of DEGs in *M*. The *p*-value is from FDR correction (FDR ≤ 0.05) and was used as a threshold. The GO terms within these conditions were considered as significantly enriched GO terms in DEGs.

#### 2.6.6. KEGG Pathway and Reactome Enrichment Analysis 

The pathway enrichment analysis identified significantly enriched metabolic or signal transduction pathways in DEGs compared with the reference whole genome using the following formula:

$$P=1-\overset{m_{-1}}{\underset{i=0}{\sum }}\frac{\left(\begin{array}{c} m\\ i \end{array}\right)\left(\begin{array}{cc} N- & M\\ n- & i \end{array}\right)}{\left[\begin{array}{c} N\\ n \end{array}\right]}$$

where “*N*” denotes the number of all transcripts within KEGG annotation, “*n*” is the number of DEGs in *N*, “*M*” is the number of all transcripts annotated without respective KEGG pathways, and “*m*” is the number of DEGs in *M*. The *p*-value is from FDR correction (FDR ≤ 0.05) and was used as a threshold. The pathways within these conditions were considered as significantly enriched KEGG pathways in DEGs. The reactome is a free online database of biological pathways [47,48]. The core unit of the reactome data model is the reaction. The nucleic acids, proteins, complexes, and small molecules that participate in reactions form a network of biological interactions and are grouped into pathways. Examples of biological pathways in the reactome include signaling, innate and acquired immune function, transcriptional regulation, translation, apoptosis, and classical intermediary metabolism. Reactome enrichment analysis identified significantly enriched reactions in DEGs compared with the whole genome background.

The same formula was used for reactome enrichment analysis.

### 2.7. Protein–Protein Interaction

Protein–protein interaction network was identified using String v10 [49], which determined genes as nodes and interaction as lines in a network. The network file was visualized using Cytoscape (v3.7.1) (Maryland, USA) software to present a core and hub gene biological interaction [50].

### 2.8. Statistical Analysis

The ANOVA was used for the statistical analysis through SAS version 8.1 software (SAS Institute Inc., Cary, NC, USA). The graphs were designed through Graph-Pad Prism6 (GraphPad, San Diego, CA, USA) software, with mean ± SEM. The variations at * *p* < 0.05 and ** *p* < 0.01 were considered to be statistically significant.

## 3. Results

### 3.1. Transfection Efficiency and Quality Evaluation of the Samples 

**Figure 1:**
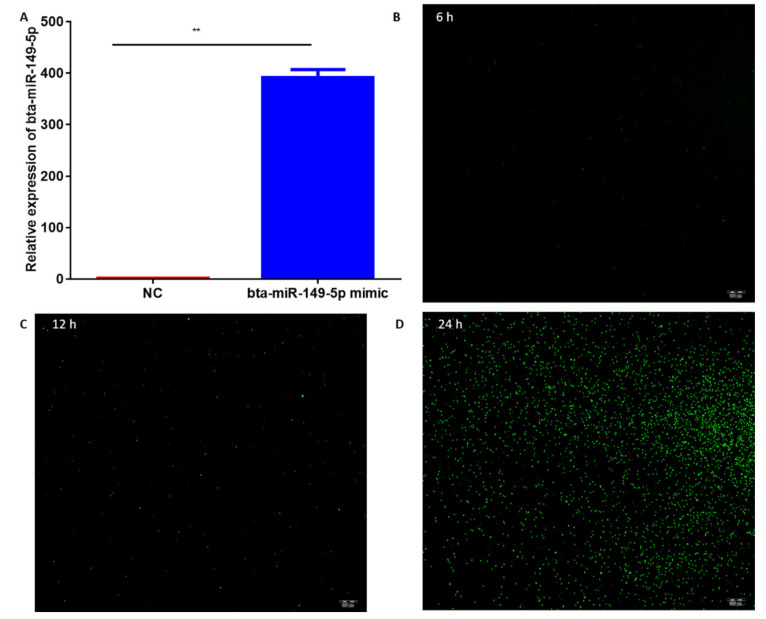
Transfection and interference efficiencies of miR-149-5p: (**A**) qPCR results showing expression levels of bta-miR-149-5p in adipocytes transfected with bta-miR-149-5p mimic and its negative control. (**B**–**D**) Transfection efficiency was checked through FAM/labeled miR-NC in bovine adipocytes; after 6-, 12-, and 24-h intervals, the transfection efficiency was measured through an Olympus I × 71 microscope (OLYMPUS) in bovine adipocytes. U6 was used as a house-keeping gene, and the relative expression level in adipocytes transfected with bta-miR-149-5p mimics was normalized with adipocytes transfected with mimic NC. The values presented with mean ± SEM, n = 3, and ** *p* < 0.01 were considered statistically significant.

To explicate regulatory function of bovine bta-miR-5p in adipogenesis, we analyzed the mimic efficiency of bta-miR-149-5p in bovine adipocytes. The expression level of bta-miR-149-5p in cells transfected with bta-miR-149-5p mimic was significantly (*p* < 0.01) increased as compared to mimic NC (Figure 1A). Before analyzing the mimic efficiency, we detected the transfection efficiency of the miRNA through FAM-labeled negative control miRNA (gene pharma, Shanghai, China). The transfection efficiency was evaluated at 6-, 12-, and 24-h intervals. The transfected adipocytes were observed under fluorescence microscope, and the photographs were captured though Olympus IX71 microscope (OLYMPUS, Tokyo, Japan) (Figure 1B–D). These findings confirmed the accuracy and success of the transfection experiment performed in the current study and authenticated the reliability of data in subsequent experiments.

**Figure 2:**
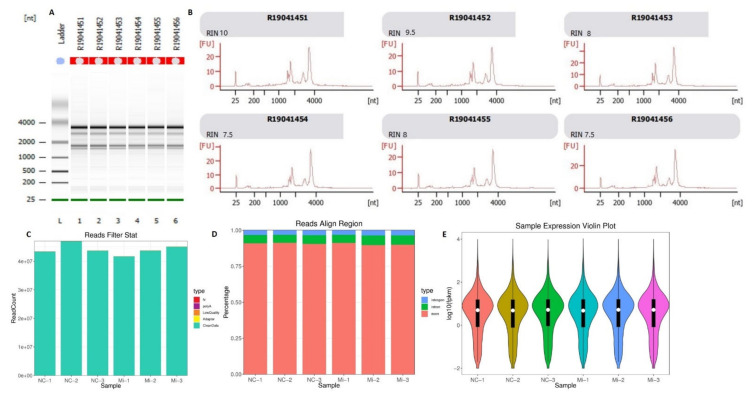
Quality and integrity of the RNA samples and data analysis. (**A**) RNA quality and integrity of adipocytes transfected with miR-149-5p mimic and mimic NC. (**B**) The reliability of the sequenced data collected from adipocytes transfected with miR-149-5p mimic and mimic NC. (**C**) The filtering of clean reads: the blue color represents the high-quality reads after removal of the adapters and low-quality bases. (**D**) The clean reads were aligned and represented with different colors, which exhibited different types of genomic regions: intergenic region (blue), intronic region (green), and exonic region (red). (**E**) The distribution of FPKM (fragment per kilobase of transcript per million mapped reads) in each sample of the 2 groups (adipocytes transfected with miR-149-5p mimic and mimic NC). The different colors represent different samples processed as mentioned below each sample.

The RNA quality shows RNA integrity number (RIN) ranged from 7.5 to 10 (Figure 2A). These figures show integrity of the RNA samples used in the current study. Moreover, sequenced clean reads were filtered (Figure 2C) and aligned with ribosome RNA (rRNA) and reference genome (Figure 2D) for purification and normality of the data for subsequent analysis. The expression abundance also confirmed the significance of the transcriptomic analysis of the samples (Figure 2E). 

### 3.2. Bta-miR-149-5p Regulated Adipogenesis

**Figure 3:**
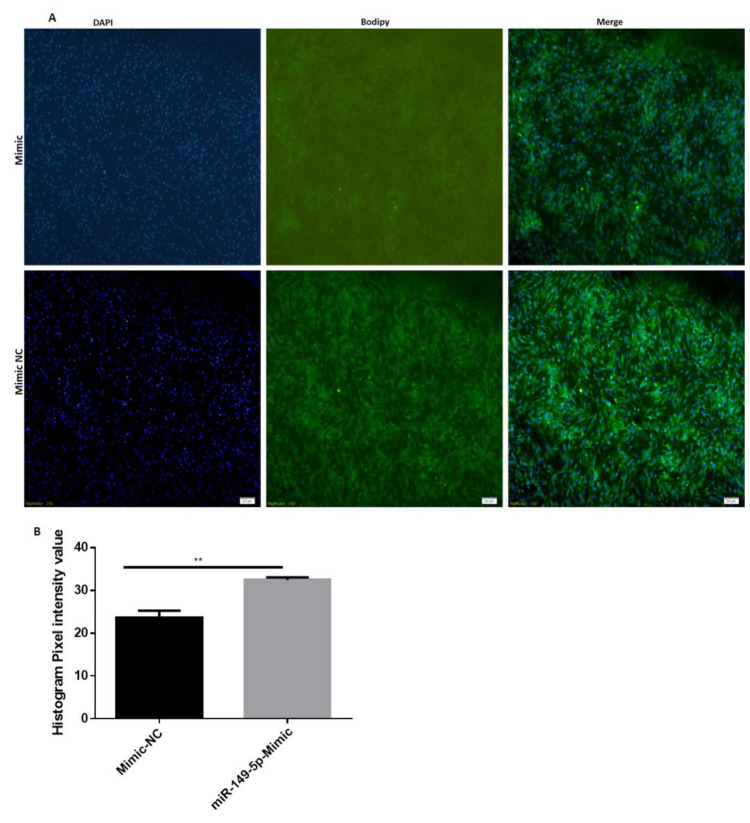
Bta-miR-149-5p represented bovine adipocyte differentiation. The photos were captured through an Olympus 1 × 71 microscope (Olympus, Tokyo, Japan). (**A**) The pictures of BODIPY-stained LDs in bovine adipocytes are represented in green, and the nuclei stained with 4, 6-diamidino-2-phenylindole (DAPI) are represented in blue. (**B**) The histogram pixel intensity values of adipocytes transfected with bta-miR-149-5p mimics or mimic NC. The values are presented with mean ± SEM, n = 3; ** *p* < 0.01 indicates statistical significance.

The lipid droplets (LDs) on day 9 of differentiation were assessed using BODIPY staining (Figure 3). The adipocytes transfected with bta-miR-149-5p mimics exhibited decreased level of LDs as compared with adipocytes transfected with mimic NC. The histogram pixel intensity also showed significant (*p* < 0.01) reduction in LDs in adipocytes transfected with bta-miR-149-5p mimics as compared with mimic NC. These results illustrate the fact that bta-miR-149-5p inhibited differentiation of bovine adipocytes.

**Figure 4:**
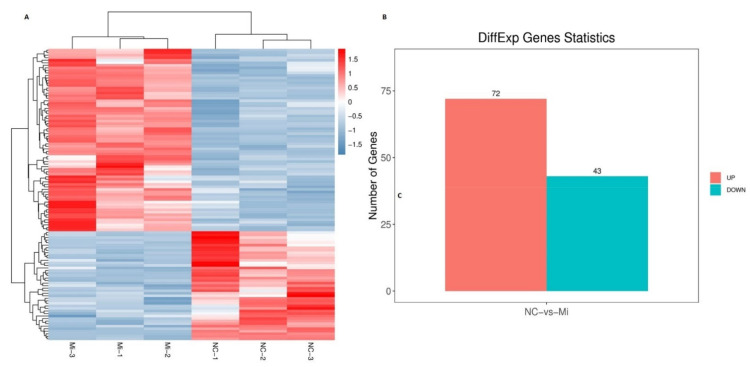
Quantification of DEGs in differentiated bovine adipocytes transfected with bta-miR-149-5p mimic and mimic NC. (**A**) The heatmap gene expression chart for the hierarchical cluster analysis of DEGs between 2 different groups of adipocytes transfected with bta-miR-149-5p mimic (n = 3) and mimic NC (n = 3). (**B**) The bar graph represents the number of DEGs between the 2 groups of differentiated bovine adipocytes transfected with bta-miR-149-5p mimic (n = 3) and mimic NC (n = 3). The red shows the number of upregulated genes and blue represents the number of downregulated genes.

Deep RNA-sequencing analysis revealed that bta-miR-149-5p significantly affected expression of adipogenic marker genes in differentiated bovine adipocytes transfected with bta-miR-149-5p mimic and mimic NC (Figure 4A–C). In total, there were 115 DEGs (differentially expressed genes), with 1.5 ± |FC and (log2 (fc) > 0.58496/log 2 (fc) > 0.58496 and *p*-value (<0.05). These 115 DEGs contain 72 upregulated and 43 downregulated genes in bovine differentiated adipocytes (Table S2). 

**Figure 5:**
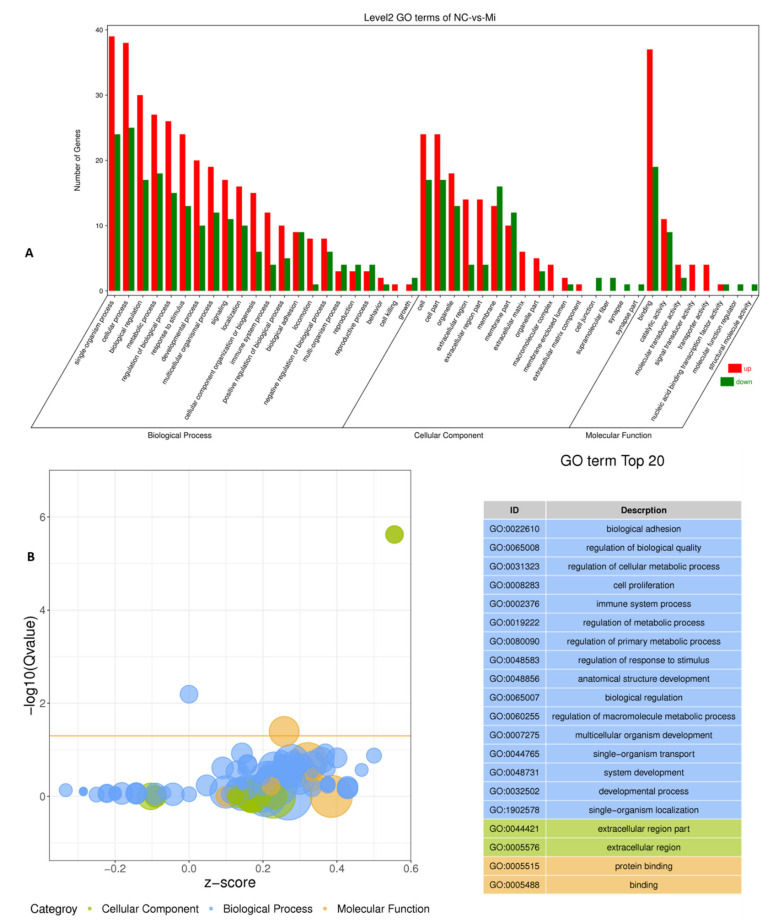
The Gene Ontology (GO) classification of DEGs in 2 groups of bovine adipocytes, transfected with bta-miR-149-5p (n = 3) mimics and mimics NC (n = 3). (**A**) GO terms of all unigenes identified in the transcriptome of differentiated bovine adipocytes within bta-miR-149-5p mimics (n = 3) and mimics NC (n = 3). The bars in each GO term were represented with green (downregulated genes) and red bars (upregulated unigenes). (**B**) The z-score within each category of GO term is represented with blue (biological process), green (cellular component), and yellow (molecular function).

The bta-miR-149-5p significantly changed the GO terms in bovine adipocyte transfected with bta-miR-149-5p mimics and mimics NC (Figure 5A). The unigenes and GO term biological processes were the most annotated unigene contributor part with 80.08%, followed by cellular component (13.4%) and molecular function (6.7%). The top 20 GO terms due to DEGs are shown in Figure 5B. The DEGs significantly altered the GO term GO:0044421 extracellular region part and GO:0005576 extracellular region in cellular component GO ontologies. The GO terms altered in molecular function ontologies in the top 20 GO terms were GO:0005515 protein binding and GO:0005488 binding. The most important GO terms altered in cellular component ontologies in the top 20 GO terms were GO:0031012 extracellular matrix, GO:0005125 cytokine activity, GO:0005102 receptor binding, GO:0016787 hydrolase activity, GO:0022610 biological adhesion, GO:0065008 regulation of biological quality, GO:0031589 cell−substrate adhesion, GO:0031323 regulation of cellular metabolic process, GO:0042326 negative regulation of phosphorylation, and GO:0042325 regulation of phosphorylation in bovine adipocytes.

**Figure 6:**
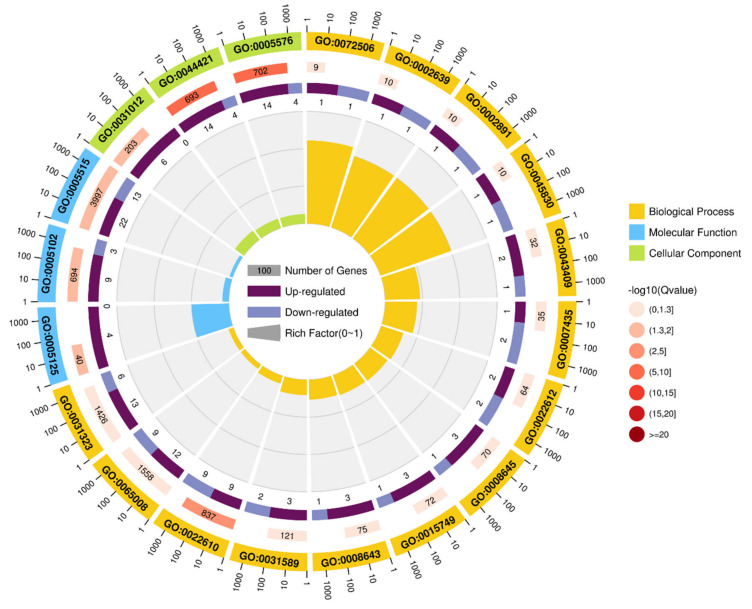
The Gene Ontology (GO) classification of DEGs in 2 groups of bovine adipocytes, transfected with bta-miR-149-5p (n = 3) mimics and mimics NC (n = 3). Each category of GO term was represented with different colors: yellow reflects biological process, green shows cellular component, and blue depicts molecular function.

Different expression trends of DEGs were found in different GO terms (Figure 6, Table S3). In GO terms of biological processes, 13 genes were upregulated and 6 were downregulated in GO:0031323 regulation of cellular metabolic process; 12 genes were upregulated and 9 were downregulated in GO:0065008 regulation of biological quality; 9 genes were upregulated and 9 were downregulated in GO:0022610 biological adhesion; and 3 genes were upregulated and 1 gene was downregulated each in GO:0031589 cell-substrate adhesion, GO:0008643 carbohydrate transport, GO:0015749 monosaccharide transport, and GO:0048645 organ formation, respectively. A single gene was up- and downregulated each in GO:0072502 cellular trivalent inorganic anion homeostasis, GO:0002639 positive regulation of immunoglobulin production, GO:0002891 positive regulation of immunoglobulin-mediated immune response, and GO:0045830-positive regulation of isotype switching. In biological function ontologies, 22 genes were upregulated and 13 were downregulated in GO:0005515 protein binding, 9 genes were upregulated and 3 were downregulated in GO:0005102 receptor binding, and all 4 genes were upregulated in GO:0005125 cytokine activity. In the category of cellular component ontologies, 14 genes were upregulated and 4 were downregulated, each in GO:0005576 extracellular region and GO:0044421 extracellular region part, respectively. All six genes were upregulated in GO:0031012 extracellular matrix.

**Figure 7:**
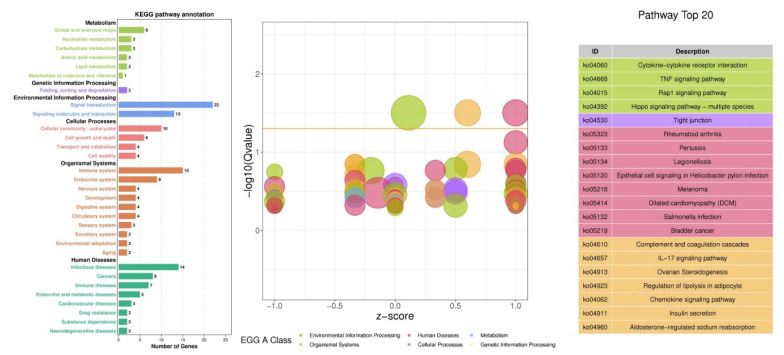
The KEGG pathway analysis of DEGs in 2 groups of bovine adipocytes transfected with bta-miR-149-5p (n = 3) mimics and mimics NC (n = 3). The KO enrichment difference bubble chart: the ordinate is -log10 (Qvalue), the abscissa is the z-score value (the difference between the number of upregulated genes and the number of downregulated genes accounts for the proportion of the total differential genes), and the yellow line represents Qvalue = 0.05 threshold. On the right is the list of pathways with the top 20 Q values. Different colors represent different A classes.

The pathway analysis of the DEGs was performed to explore the pathways and molecular interactions in bovine adipocytes transfected with bta-miR-149-5p mimics or mimics NC. The top 20 significantly enriched KEGG pathways, based on the Q < 0.05 value calculated by hypergeometric test, demonstrated the roles of DEGs in adipocyte differentiation (Figure 7). DEGs altered the top KEGG_A_class enrichment pathways including metabolism (global and overview maps, nucleotide, amino acids, carbohydrates, and lipid metabolism), genetic information processes (signal transduction, signaling molecules, and interaction), cellular process (cellular community eukaryotes, cell growth and death, transport and catabolism, cell motility, cell motility), and organismal systems (immune system, endocrine system, nervous system, development, digestive system, circulatory system); under diseases, it also regulated infectious diseases, cancers, immune diseases, endocrine and metabolic diseases, and cardiovascular diseases. These KEGG_A class pathways perform crucial roles in adipogenesis.

The top 20 pathways affected by bta-miR-149-5p through DEGs within differentiated bovine adipocytes were ko04060 cytokine−cytokine receptor interaction, ko04668 TNF signaling pathway, ko04015 Rap1 signaling pathway, ko04392 Hippo signaling pathway—multiple species, ko04530 tight junction, diseases, ko04923 regulation of lipolysis in adipocyte, ko04062 chemokine signaling pathway, and ko04911 insulin secretion.

**Figure 8:**
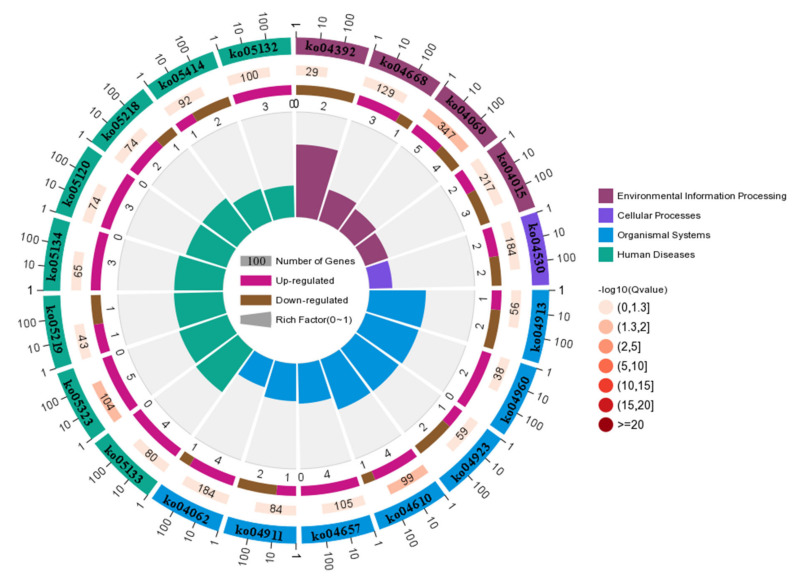
KO enrichment circle diagram of the KEGG_A_class: The first circle: the first 20 pathways of enrichment; outside the circle is the coordinate ruler of the number of genes. Different colors represent different A classes. The second circle: the number of pathways in the background genes and the Q value. The more genes, the longer the bar, the smaller the Q value, the redder the color. The third circle: the bar graph of upregulated gene ratio, wherein dark purple represents the upregulated gene ratio and light purple represents the downregulated gene ratio; the specific value is shown below. The four circles: Rich factor value of each pathway (the number of differences in the pathway divided by all the numbers); background grid lines each grid represent 0.1.

The different KEGG_A_class pathways were significantly altered in adipocytes transfected with bta-miR-149-5p mimics and its negative control NC (Figure 8, Table 1 and Table S4). The highly effected KEGG_A_class was the human disease that altered the most important KEGG_B_class pathways including immune diseases, infectious diseases, cardiovascular diseases, and cancers. The second most affected KEGG_A_class pathway was organismal systems, which significantly altered the KEGG_B_class pathways including immune systems, endocrine system, and excretory system; the most important corresponding pathways altered by these KEGG_B class were IL-17 signaling pathway, regulation of lipolysis in adipocyte, chemokine signaling pathway, insulin secretion, and aldosterone-regulated sodium reabsorption. The role of these pathways in adipogenesis is well documented. The environmental information processing and cellular processes were also the most significantly changed KEGG_A_class pathways by bta-miR-149-5p in bovine adipocytes, the former changed signal transduction, signaling molecules, and interaction, and the latter changed cell growth and death, cell motility, and cellular community—eukaryotes KEGG_B_class pathways. These KEGG_B classes altered the most important pathways, including TNF signaling pathway, Hippo signaling pathway—multiple species, Rap1 signaling pathway, tight junction, and focal adhesion. Moreover, metabolism was also found to be one of the mostly affected pathways in the KEGG_A_class, and the KEGG_B_class pathways in this category included lipid metabolism, carbohydrate metabolism, amino acid metabolism, metabolism of cofactors and vitamins, nucleotide metabolism, and global and overview maps.

**Figure 9:**
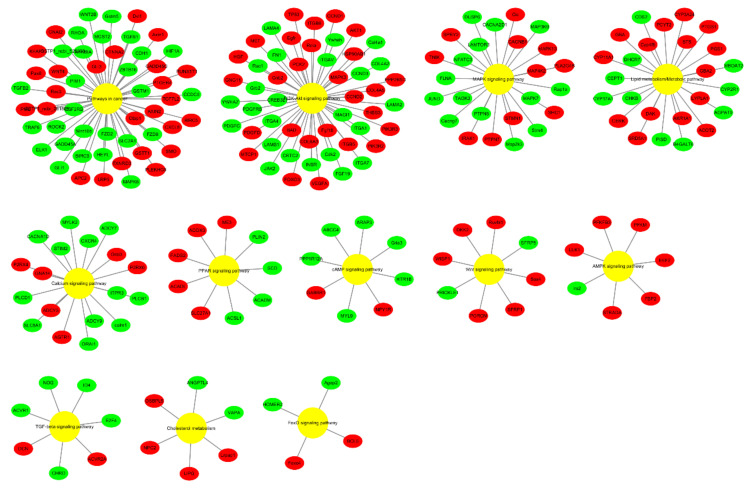
The distribution of up- and downregulated DEG enrichment in different KEGG (Kyoto Encyclopedia of Genes and Genomes) pathways. The pathway nodes are represented by pathways (yellow), downregulated genes (green), and upregulated genes (red), which are shown as circles.

Moreover, we explored the relationship between the DEGs and signaling pathways through Cytoscape software (Figure 9). The KEGG pathways regulated by these DEGs were PI3K-Akt signaling pathway, calcium signaling pathway, pathways in cancer, MAPK signaling pathway, lipid metabolism/metabolic pathway, PPAR signaling pathway, AMPK signaling pathway, TGF-beta signaling pathway, cAMP signaling pathway, cholesterol metabolism, Wnt signaling pathway, and FoxO signaling pathway. Furthermore, the expression trend of the DEGs in these pathways were also exploited. These data indicate the role of bta-miR-149-5p in adipogenesis through interactive regulation of DEGs and their respective KEGG pathways in bovine adipocytes. 

**Figure 10:**
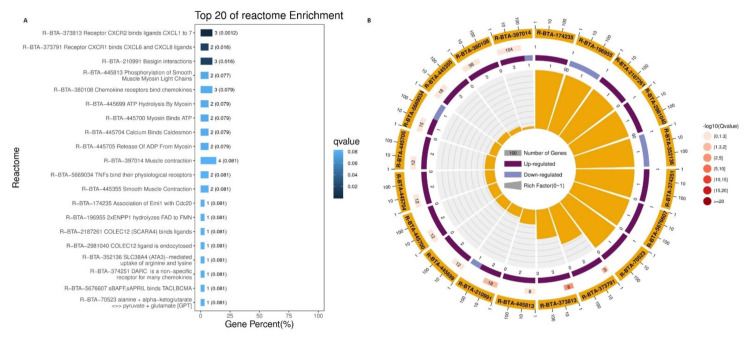
Top 20 reacrome enrichment pathways. (**A**) Reactome enrichment bar graph: the top 20 reactome channels with the smallest Q value to plot; the ordinate is the reactome channel, and the abscissa is the percentage of the number of reactome channels in all the DEGs. The darker the color, the smaller the Q value. The value on the column is the number of reactome channels and the Q value. (**B**) Reactome enrichment circle diagram: the first circle: the top 20 reactome pathways of the enrichment; outside the circle is the coordinate ruler of the number of genes. The second circle: the number of the reactome pathway in the background gene and the Q value. The more the genes, the longer the bars, the smaller the Q value, and the redder the color. The third circle: the bar graph of upregulated gene ratio, wherein dark purple represents the upregulated gene ratio and light purple represents the downregulated gene ratio; the specific value is shown below. The fourth circle: each reactome pathway Rich factor value (the number of differences in the reactome channel divided by all the numbers); in terms of background grid lines, each grid represents 0.1.

In addition to this, reactome biological pathways were also studied in terms of the DEGs. As shown in Figure 10, the most important pathways in top 20 reactome enrichment pathways were R−BTA−373813 receptor CXCR2 binding ligands CXCL1 to 7, R−BTA−373791 receptor CXCR1 binding CXCL6 and CXCL8 ligands, R−BTA−210991 basigin interactions, R−BTA−380108 chemokine receptors binding chemokines, R−BTA−445699, ATP hydrolysis by myosin, R−BTA−445704 calcium binding caldesmon, and R−BTA−5669034 TNFs binding their physiological receptors. These reactome pathways play crucial roles in adipogenesis. 

### 3.3. Validation and Identification of Key DEGs during Adipogenesis through qRT-PCR

**Figure 11:**
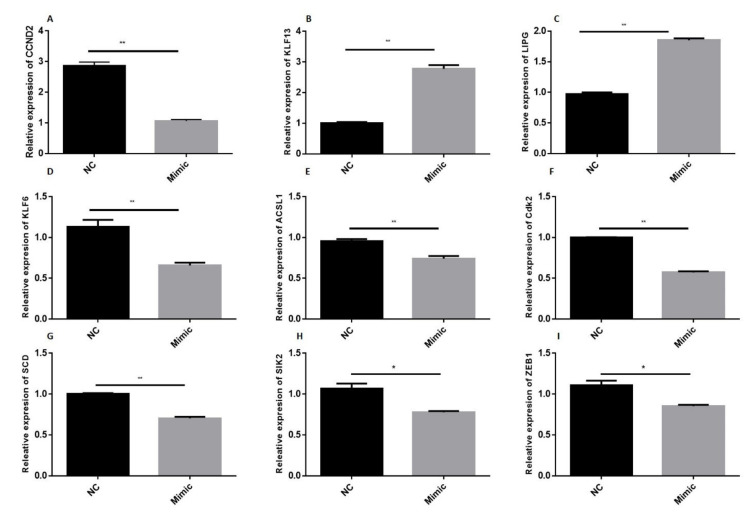
Validation of RNA-Seq data through qRT-PCR. Expression (**A**–**I**) relative mRNA levels of CCND2, KLF12, LIPG, KLF6, ACSL1, Cdk2, SCD, SIK2, and ZEB1 in bovine adipocytes transfected with bta-miR-149-5p mimics or mimics negative control (NC). The values exhibit the mean ± SEM (n = 3), and statistical significance is shown by * *p* < 0.05 and ** *p* < 0.01.

The RNA sequence results were validated through qRT-PCR analysis of key marker genes selected from the list of DEGs (Figure 11). The mRNA levels of CCND2, KLF6, ACSL1 Cdk2, SCD, SIK2, and ZEB1 were significantly (*p* < 0.01) downregulated in adipocytes transfected with bta-miR-149-5p mimics. However, upregulation of bta-miR-149-5p significantly (*p* < 0.01) enriched relative mRNA expression of KLF12 and LIPG genes in bovine adipocytes. These results are in line with the results of sequencing data.

## 4. Discussion

Improvement of intramuscular fat is a challenge for the experts of animal science for the improvement of meat quality traits, and obesity is a global issue—a root cause of certain fatal human disorders [51]. Adipogenesis is a complex biochemical process, characterized by adipocyte proliferation, maturation, and differentiation of preadipocytes. The preadipocytes that are derived from the existing pool of adipocytes differentiate in response to proper signals [52]. Therefore, it is imperative to illuminate the underlying molecular mechanism of adipogenesis. In the recent past, a series of studies proved the crucial functions of microRNAs in the proliferation, differentiation, and function of adipocytes. High-throughput sequencing and microarray analysis revealed the roles of novel miRNAs in animals [53]. Previously, the roles of let-7 [54], miR-21a [55], miR-23a [56,57], miR-27a/b [58,59], miR-130 [60], miR-185 [61], miR-448 [62], and miR-709 [63] in lipogenesis negatively regulated adipocyte differentiation, whereas miR-21 [64,65], miR-26b [66], miR-143 [67], miR-148 [68], miR-183 [69], miR-424 [14], and miR-425 [70] positively regulated adipocyte differentiation. Previously, we confirmed the role of bta-miR-149-5p bovine adipocyte proliferation and differentiation [32]. These findings imply that bta-miR-149-5p is a novel hallmark for dealing with the problem of low intramuscular fat in cattle breeding programs.

In the present study, deep RNA-sequencing analysis revealed that bta-miR-149-5p significantly affected expression of adipogenic marker genes in differentiated bovine adipocytes transfected with bta-miR-149-5p mimic and mimic NC. The unigenes and GO term biological processes were the most annotated unigene contributor part with 80.08%, followed by cellular component of 13.4%, and molecular function of 6.7%. Interestingly, in the present study, the DEGs were upregulated in all three GO categories, namely, in biological processes, biological function, and cellular component ontologies. These findings show that bta-miR-149-5p regulates the functional roles of DEGs in bovine adipocytes through regulation of GO terms. Furthermore, the pathway analysis of the DEGs was performed to exhibit the pathways and molecular interactions in bovine adipocytes transfected with bta-miR-149-5p mimics or mimics NC. Strikingly, the pathway enrichment analysis explored the KEGG_A_classes, including disease and metabolism regulated by the DEGs in adipocytes transfected with bta-miR-149-5p, which specifically categorized the most important KEGG_B class pathways with proven roles in metabolism and adipogenesis. The pathway-based studies helped to exploit the gene roles in biological functions [46,71]. The adipocytes perform various functions, particularly energy storage in the form of triglycerides [72]. Adipose is also an endocrine organ, secreting various bioactive molecules such as adipokines, which regulate metabolic functions in body system. Recently, intensive research has been conducted to show miRNA’s potential regulatory roles in metabolism [73]. Moreover, the expression trend of DEGs in these pathways exhibited an upregulated trend, and the number of regulated DEGs were more than the number of downregulated genes in these pathways.

In addition to this, the specific KEGG pathways regulated by these DEGs were PI3K-Akt signaling pathway, calcium signaling pathway, pathways in cancer, MAPK signaling pathway, lipid metabolism/metabolic pathway, cholesterol metabolism, PPAR signaling pathway, AMPK signaling pathway, TGF-beta signaling pathway, cAMP signaling pathway, Wnt signaling pathway, and FoxO signaling pathway. These data indicate the role of bta-miR-149-5p in adipogenesis through interactive regulation of DEGs and their respective KEGG pathways in bovine adipocytes. All these pathways have proven roles in adipogenesis. Previously, it has been reported that PI3K-Akt signaling pathway plays an important role in adipose tissue function and energy metabolism through regulation of the genes expressed in lipid metabolism [74,75,76]. Previous literature has shown the association of FoxO signaling, PI3K-Akt signaling, and cAMP signaling pathways with lipid metabolism [77,78,79]. The cAMP and calcium signaling pathways act as the second messengers in eukaryotic cells [80] and regulate the intracellular lipolysis in adipocytes through calcium ions and cAMP levels [81]. There is substantial evidence that MAPK signaling pathway [82,83], lipid metabolism, insulin signaling [84], AMPK signaling pathway, Wnt signaling pathway [85,86,87,88], and TGF-beta signaling pathway [89,90] perform crucial roles in adipogenesis. The results of the present study support the interactive roles of the signaling pathways in the regulation of adipogenesis.

Interestingly, the pathway enrichment analysis in the present study also exhibited the role of bta-miR-149-5p in cancer. Previously, the roles of miR-149 in osteosarcoma [29], cancers of the digestive system [21], colorectal cancer [22], hepatocellular cancer [23,24], respiratory system cancer [25], breast cancer [26], and sebaceous gland carcinoma of the eyelid [27] have been well documented. Furthermore, in the present study, overexpression of bta-miR-149-5p significantly (*p* < 0.05) upregulated *LIPG* (*endothelial lipase*) and *KLF12* expression, which perform vital roles in cancer [91,92]. However, cancer study is beyond the scope of the present study, yet there is a very close relation of adipocytes and cancers. Adipocytes are the primary stromal cells, performing a dynamic role in the cancer microenvironment. Cancer-associated adipocytes (CAAs) are found next to the cancer cells and communicate with the tumor cells via secreting several factors that mediate local and systemic effects. Recently, miRNAs have been considered mediators of intercellular communication in the cancer microenvironment. Tumor cells coordinate with stromal cells and other extracellular matrix (ECM) elements and establish a comfortable niche to grow in in order to escape the immune system. Inside the cancer microenvironment of the cells, the miRNAs influence and take over the physiological processes of surrounding cells and thus promote cancer progression [93]. However, these findings need further research in order to fully explore this molecular mechanism of microRNA in cancer-associated adipocytes (CAAs). 

Moreover, the bta-miR-149-5p significantly (*p* < 0.01) downregulated the mRNA levels of adipogenic marker genes such as CCND2, KLF6, ACSL1, Cdk2, SCD, SIK2, and ZEB1 in bovine adipocytes. These findings validated the transcriptomic data and also proved the functional roles of bta-miR-149-5p in bovine adipogenesis through regulation of these adipogenic marker genes’ expression. The cyclin D2 (CCND2) and Cdk2 are well-known proliferation marker genes, enriched in early differentiation and then again overexpressed in the later stage of adipocyte differentiation [94,95]. Kruppel-like factor 6 (KLF6) is a transcription factor and is a potent regulator of adipocyte differentiation [96]. ACSL1 is a proven mediator of lipid metabolism, cellular lipid content, and insulin sensitivity in adipocyte differentiation [97]. The crucial role of the SCD gene is well established in the differentiation adipocyte [98] and fatty acid composition during adipogenesis [99]. SIK2 plays a critical role in the regulation of lipid homeostasis and adipogenesis via cAMP signaling pathway [100]. Previously, our research group validated the role of ZEB1 transcription factor in bovine adipocytes, with it found to be potential regulatory motif in the promoter of an adipogenic marker gene (ABHD5) that plays vital role in bovine adipogenesis [101]. These results of qRT-PCR are in line with the results of RNA-Seq data, which further validate and authenticate the present study. Taken together, these findings suggest that the DEGs in the present study play pivotal roles in bovine adipogenesis. Generally, the present study provides valuable evidence for expanding our understanding about the molecular mechanism of bta-miR-149-5p in the regulation of lipid metabolism and adipogenesis.

## 5. Conclusions

These data indicate the role of bta-miR-149-5p in adipogenesis through interactive regulation of DEGs and their respective KEGG pathways in bovine adipocytes. In conclusion, our results suggest that bta-miR-149-5p regulates lipid metabolism in bovine adipocytes. The results of this study provide a basis for studying the function and molecular mechanism of the bta-miR-149-5p in regulating adipogenesis.

## Figures and Tables

**Table 1:** Description of KEGG pathways classes altered by DEGs in bovine adipocytes transfected with bta-miR-149-5p mimics or mimics NC.

KEGG_A_Class	KEGG_B_Class	Pathway	DEGs	Pvalue	Qvalue	Pathway ID
Environmental information processing	Signaling molecules and interaction	Cytokine–cytokine receptor interaction	9	0.000393	0.031419	ko04060
	Signal transduction	TNF signaling pathway	4	0.010095	0.164541	ko04668
	Signal transduction	Hippo signaling pathway—multiple species	2	0.015418	0.179513	ko04392
	Signal transduction	Rap1 signaling pathway	5	0.013727	0.172113	ko04015
Organismal systems	Immune system	Complement and coagulation cascades	5	0.000461	0.031419	ko04610
	Immune system	IL-17 signaling pathway	4	0.004923	0.143156	ko04657
	Endocrine system	Ovarian steroidogenesis	3	0.005853	0.143156	ko04913
	Endocrine system	Regulation of lipolysis in adipocyte	3	0.00677	0.143156	ko04923
	Immune system	Chemokine signaling pathway	5	0.007026	0.143156	ko04062
	Endocrine system	Insulin secretion	3	0.01768	0.192128	ko04911
	Excretory system	Aldosterone-regulated sodium reabsorption	2	0.025711	0.24652	ko04960
Human diseases	Immune diseases	Rheumatoid arthritis	5	0.000578	0.031419	ko05323
	Infectious diseases	Pertussis	4	0.001839	0.074941	ko05133
	Infectious diseases	Legionellosis	3	0.008847	0.160228	ko05134
	Infectious diseases	Epithelial cell signaling in *Helicobacter pylori* infection	3	0.012595	0.171085	ko05120
	Cancers	Melanoma	3	0.012595	0.171085	ko05218
	Cardiovascular diseases	Dilated cardiomyopathy (DCM)	3	0.022461	0.228825	ko05414
	Infectious diseases	Salmonella infection	3	0.027881	0.252474	ko05132
	Cancers	Bladder cancer	2	0.032344	0.26341	ko05219
Cellular processes	Cellular community—eukaryotes	Tight junction	4	0.032526	0.26341	ko04530
	Cell growth and death	Oocyte meiosis	3	0.045115	0.301575	ko04114
	Cellular community—eukaryotes	Focal adhesion	4	0.047541	0.301575	ko04510

KEGG: Kyoto Encyclopedia of Genes and Genomes. DEG: Deferentially Expressed Genes.

## Data Availability

All data are available with the manuscript as supplementary files.

## Supplementary Materials

The following are available online at https://www.mdpi.com/article/10.3390/ani11051207/s1, Tables S1–S4.
