# Supplementary material for: RNA-Seq Reveals Function of Bta-miR-149-5p in the Regulation of Bovine Adipocyte Differentiation
Source: Animals (Basel). 2021 Apr 22;11(5):1207. doi: 10.3390/ani11051207 (PMC8145242; doi:10.3390/ani11051207)
Supplement: Supplementary file 1 [file animals-11-01207-s001.zip › S1 primers.pdf]

Supplementary Table 1 Primers used in quantitative PCR

Gene	Sequence （5 '→3 '）
SCD - Forward	ACCATGAAGTGTGTCCACCA
SCD - Reverse	CCATGGCAATGAGTTTATGC
CCND2 - Forward	GGGCAAGTTGAAATGGAA
CCND2 - Reverse	TCATCGACGGCGGGTAC
GAPDH - Forward	CCAACGTGTCTGTTGTGGAT
GAPDH - forward	CTGCTTCACCACCTTCTTGA
CDK2 - Forward	GGGTCCCTGTTCGTACTTATAC
CDK2 - Reverse	CCACTGCTGTGGAGTAGTATTT
GAPDH - Forward	CCAACGTGTCTGTTGTGGAT
GAPDH - forward	CTGCTTCACCACCTTCTTGA
ACSL1 Forward	CTTCGCAGTGGCATCATTAG
ACSL1 Reverse	GTCCGTAGCCTTCGTAGAAC
β-actin Forward	CATCGGCAATGAGCGGTTCC
β-actin Reverse	CCGTGTTGGCGTAGAGGTC
ZEB1-Forward	CCGGAGATGGCAGTTTGTCT
ZEB1-Reverse	AGGTTCACGGAATCGGCAAT
